# 6G in medical robotics: development of network allocation strategies for a telerobotic examination system

**DOI:** 10.1007/s11548-024-03260-6

**Published:** 2024-09-09

**Authors:** Sven Kolb, Andrew Madden, Nicolai Kröger, Fidan Mehmeti, Franziska Jurosch, Lukas Bernhard, Wolfgang Kellerer, Dirk Wilhelm

**Affiliations:** 1 TUM School of Medicine and Health, Klinikum rechts der Isar, Research Group MITI, Technical University of Munich, Munich, Germany; 2 TUM School of Computation, Information and Technology, Chair of Communication Networks, Technical University of Munich, Munich, Germany; 3 Department of Surgery, TUM School of Medicine and Health, Klinikum Rechts der Isar, Technical University of Munich, Munich, Germany; 4 Munich Institute of Robotics and Machine Intelligence, Technical University of Munich, Munich, Germany

**Keywords:** 6G, Medical robotics, Telemedicine, Fog computing

## Abstract

**Purpose:**

Healthcare systems around the world are increasingly facing severe challenges due to problems such as staff shortage, changing demographics and the reliance on an often strongly human-dependent environment. One approach aiming to address these issues is the development of new telemedicine applications. The currently researched network standard 6G promises to deliver many new features which could be beneficial to leverage the full potential of emerging telemedical solutions and overcome the limitations of current network standards.

**Methods:**

We developed a telerobotic examination system with a distributed robot control infrastructure to investigate the benefits and challenges of distributed computing scenarios, such as fog computing, in medical applications. We investigate different software configurations for which we characterize the network traffic and computational loads and subsequently establish network allocation strategies for different types of modular application functions (MAFs).

**Results:**

The results indicate a high variability in the usage profiles of these MAFs, both in terms of computational load and networking behavior, which in turn allows the development of allocation strategies for different types of MAFs according to their requirements. Furthermore, the results provide a strong basis for further exploration of distributed computing scenarios in medical robotics.

**Conclusion:**

This work lays the foundation for the development of medical robotic applications using 6G network architectures and distributed computing scenarios, such as fog computing. In the future, we plan to investigate the capability to dynamically shift MAFs within the network based on current situational demand, which could help to further optimize the performance of network-based medical applications and play a role in addressing the increasingly critical challenges in healthcare.

## Introduction

Healthcare systems around the world are increasingly facing severe challenges due to a multitude of problems such as staff shortage, changing demographics, and the reliance on an often strongly human-dependent environment [1]. One approach aiming to address these issues is the development of new telemedicine applications. While the term telemedicine is quite broad, encompassing all types of healthcare provided to a patient at a distance, the modern concept of telemedicine was revolutionized by the advent of digital telecommunication [2]. In recent years, medical applications in this field have evolved from pure teleconferencing solutions to more advanced applications such as telemedical clinical examinations and even telesurgery [3].

These emerging systems place stringent requirements on the communication network infrastructure in terms of reliability, bandwidth, latency and processing power [4]. To satisfy these demands, the underlying network needs the capability to flexibly and dynamically manage a wide range of applications with different requirements. This approach is currently being researched within the new communication standard 6G, which is envisioned to be the “network of everything,” combining technologies introduced in 5G with new architectures and functions [5, 6].

To explore the potential of incorporating such new network concepts in telemedical applications, we developed a distributed robot control infrastructure for a multimodal telemedical platform. In this paper, we use this system to investigate the benefits and challenges of offloading application components to optimize the performance of the telemedical platform. Using a simplified control pipeline, we examine different task distributions for which we characterize the network traffic and computational loads. We subsequently establish network allocation strategies for different types of tasks with varying requirements, which will benefit the development of medical robotic applications using distributed computing scenarios to optimize performance.

The remainder of this paper is organized as follows: In Section “Related work”, we give an overview of the state of the art of the relevant research areas. In Section “Methods", we describe the envisioned 6G radio access network (RAN) architecture, the overall system architecture as well as the demonstration task used for evaluation and close with an overview of the evaluation procedure. In Section “Results", we show the evaluation results of our system setup, which are subsequently discussed in Section “Discussion". Finally, we provide some insights on how we will expand on the investigation of our system setup in future work.

## Related work

The main focus of telemedical clinical examinations has so far been on specific, single-modal applications such as robotic ultrasound or robotic auscultation systems [7, 8]. Robotic ultrasound systems range from fully teleoperated to fully autonomous approaches, although they have not yet reached a maturity level sufficient for clinical use [7]. Current challenges include the improvement of performance by increasing system autonomy, the improvement of reproducibility and safety, and the integration of multimodal imaging [7, 9]. Furthermore, several first approaches for autonomous auscultation systems have been presented in the past years. However, these generally focus on specific examination procedures, such as cardiac and/or pulmonary examinations [10–12]. To offer a diagnostic spectrum comparable to a face-to-face examination, it is necessary to develop multimodal systems which integrate different diagnostic methods (e.g., ultrasound, auscultation, palpation, etc.) into one application [8, 13]. A direct consequence of this approach is that the integration of multiple modalities, the increase in autonomy and the integration of more information with higher data quality causes higher computational loads as well as stronger requirements on the network infrastructure in terms of latency and bandwidth.

To leverage the potential of these multimodal systems, it is necessary to overcome the limitations of current networks. The network standard 6G promises to deliver many new features that could enable the development of such telemedical applications [4]. Aside from lower latencies, higher available bandwidths, higher system reliability, and new use cases to integrate new forms of machine-to-machine (M2M) communication, the envisioned convergence of computing and networking resources to a single computing continuum has strong potential to enhance telemedical applications [14]. Since the emergence of cloud computing in the late 1990s, the idea of distributed computing has evolved to integrate many new concepts such as edge computing, fog computing and in network computing [15–17]. While edge computing considers computing resources located at the network edge, in network computing is based on the idea of executing applications on network elements such as (programmable) switches and routers [15, 17]. Intelligent edge computing (IEC) in 5G aims to further reduce the latency and network traffic between devices and the cloud computing resources by considering the distance of network resources to the user [18]. In a somewhat more general approach, the fog computing paradigm aims to extend cloud computing to the network edge by providing compute, storage, and networking resources between user equipment (UE) and a centralized cloud [16, 19]. As mentioned above, 6G aims to merge computing and networking resources to a single continuum, simultaneously reducing the hardware requirements at user endpoints while increasing the flexibility and reliability of the system through dynamic resource allocation strategies [14, 18].

In this work, we show how such allocation strategies can be developed by examining the network characteristics of different types of tasks with varying requirements. Note that we refer to our distributed computing architecture as fog computing throughout the rest of the paper, since the definition given in [16] fits best to the approach presented in this work.

## Methods

### Envisioned 6G RAN architecture

**Fig. 1:**
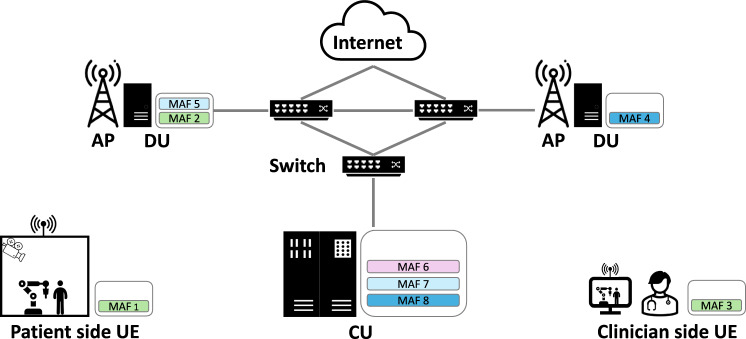
Envisioned 6G RAN architecture to provide wireless connectivity for various medical use cases. It consists of access points (APs), distributed units (DUs), central units (CUs), an access path to the Internet and interconnecting network switches. The architecture allows modular application functions (MAFs), i.e., parts of medical applications, to be executed on local user equipment (UE) or on network resources

A 6G network is envisioned to consist of new concepts and technologies, such as the native integration of machine learning (ML) and artificial intelligence (AI) [6, 14]. However, it also aims to merge and integrate existing networks and architectures [14]. In contrast to previous networking generations, the interaction between application computing and networking will be much closer in 6G, leveraging not only placement algorithms in the network using ML/AI or numerical approaches, but also computational capabilities of general-purpose processors and programmable network elements via concepts such as software-defined networking (SDN) [20]. In the following, we describe such a 6G network using wireless access technology. It consists of a RAN and a core network. While the former connects the user equipment (UE) to the network, the latter is responsible for forwarding information within the Internet. In this concept, the RAN consists of several components, as shown in Fig. 1. UEs such as smartphones, robots or medical devices can physically communicate with access points (APs) using technologies such as 5G/6G base stations, LiFi, or WiFi. Furthermore, processing units placed within the network, either as a central unit (CU) or distributed unit (DU), enable the development of distributed computing scenarios for applications. DUs are typically located closer to the AP and thus to the UE, which consequently leads to reduced latency. However, DUs are usually limited in their processing capabilities. CUs, on the other hand, are usually more capable of processing computationally intensive applications but are located further away from the APs and UEs, adding considerable delays that may violate the latency requirement of time-sensitive tasks. These computing capabilities do not only allow the dynamic distribution of network functions (i.e., functions necessary for the network connection, for optimal network performance) but also parts of applications, so-called modular application functions (MAFs). MAF is a generalized term and can represent a software component of any type of application which can be executed either on local or network resources. The main advantage of this generalization is the optimized placement potential. By handling abstract MAFs with various attributes such as priority, throughput constraints, and latency constraints, the actual executed application is hidden for the placement within the network, allowing any type of application (including network functions) to be placed according to the desired optimization parameters. As an example, in Fig. 1 a robotic examination suite is shown on the patient side, which is operated by a doctor on the clinician side. In this case, several MAFs are needed to perform various tasks such as robot controlling, robot path planning, video and audio streaming. To further improve the quality of service (QoS) of such applications, the interaction between the network and MAFs can be considered during development. The advantage of using MAFs is that they can be dynamically placed and executed on different processing units depending on the current state of the network. That way, the overall performance of an application and the whole network (number of executed applications, power consumption, etc.) can be improved even further. All components are interconnected with networking switches, which can be programmable, enabling the packet forwarding behavior to be completely defined by the network operator. Finally, the RAN is connected to the Internet via a core network.

### System architecture

**Fig. 2:**
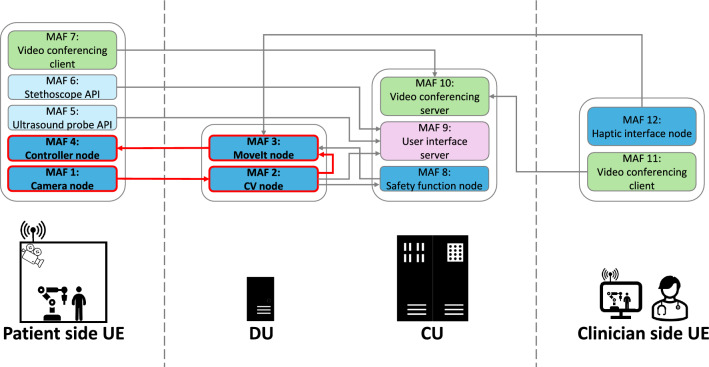
Flowchart laying out the distributed control system architecture using the MAF concept. The arrows indicate data being passed from one MAF to another. The demonstration task pipeline (MAF 1-4)—as described in Section “Demonstration task"—is highlighted in red

The system architecture of our distributed robotic control is depicted in Fig. 2. It follows the network topology shown in Fig. 1 by distributing components between the patient side, clinician side, and resources within the network. The control architecture was developed using ROS 2, given its advantages in distributed setups due to the data distribution service (DDS) communication middleware [21]. The implementation uses FogROS2, a package which allows the robot programmer to delegate any ROS node to be launched on a virtual server from various cloud providers [22]. FogROS2 handles instantiating the server, setting up necessary dependencies for the robot application, and configuring a virtual private network (VPN) between the nodes to enable communication. The application was fully containerized to enable it to run remotely within a data center context and be completely platform-agnostic.

**Fig. 3:**
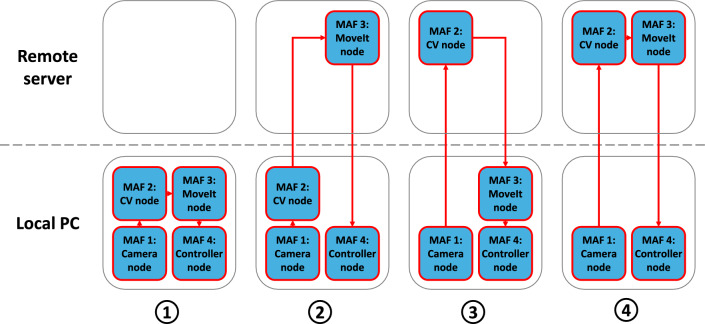
The system configurations investigated in this work. MAFs were delegated to either the local or remote computer at launch time by editing the launch file for the demonstration task

To ensure that all processes could meet the real-time requirements of the robotic controller, the configuration of the Docker container was adapted to give the containerized applications the same capabilities as if they were natively running on the host machine. To make the containerization as transparent as possible to the processes within, the container was configured to start with the SYS_NICE capability, which allows the container to change the niceness of processes, set real-time scheduling policies, CPU affinity, and other operations [23]. Furthermore, to allow the nodes in the docker container to communicate with the remote nodes hosted on the server and with the robot controller, all virtual networking performed by both Docker and the orchestrator software was disabled by configuring the container to exclusively use the host network. We used K3s, a lightweight version of Kubernetes, as the orchestration solution to manage the Docker containers due to its focus on performance and easy setup [24]. For evaluation purposes, the demonstration task described in the following section was integrated in a model setup consisting of a local, consumer-grade PC (Dell Precision 3240, Intel Core i7-10700) on the patient side and a performance-oriented remote server (Dell 3930, Intel Xeon E-2286G) as a DU. The computers were linked via a cable-based private subnetwork using a network switch (Ubiquiti Enterprise XG 24).

### Demonstration task

To provide a basis for the investigation of the robotic control infrastructure, a subset of the modular system was used as a demonstration task. The pipeline for this demonstration task is highlighted in Fig. 2. The task was chosen to include all components needed to approximate a basic robotic workflow. Furthermore, the included components were specifically selected to illustrate the diverse requirements of MAFs. In this case, the simplified workflow was defined as the autonomous placing of a diagnostic instrument, such as a stethoscope or ultrasound probe, onto the abdomen of a patient at a defined contact point. Consequently, the high-level strategy of the demonstration task was to visually identify a target point via one of the integrated cameras, and then navigate the tool center point (TCP) of the robot arm to this point in a predefined orientation.

In the first step, the camera node (MAF 1) brings images streamed from a depth camera (RealSense D405, *Intel Corporation*, USA) attached to the robotic arm (Panda, *Franka Emika GmbH*, Germany) into the ROS 2 ecosystem via the RealSense Software Development Kit (SDK) and published as a ROS 2 message [25]. While many configuration parameters exist, the default settings were used in this evaluation to keep the setup as general as possible. The computer vision (CV) node (MAF 2) is then used to identify the target point in the received images. The target for this task is an ArUco marker placed on the examination table [26]. Given their predetermined structure, the identification and pose extraction of these markers is very straightforward. In this setup, the target identification is performed using OpenCV [27]. Once the target pose is determined relative to the camera’s coordinate frame, it is converted into a pose relative to the robot base coordinate frame using the tf2 library [28]. With the target representation in the correct coordinate frame, the trajectory is generated by the path planning node (MAF 3) via the MoveIt 2 framework, an open-source robotic manipulation platform [29]. A rapidly exploring random tree planner (RRT-Connect) is used to generate and validate the trajectory, as it is fast and sufficiently accurate for an initial characterization. The final step in the demonstration task pipeline is to execute the planned trajectory via the local controller (MAF 4). Each of these steps is performed by a separate ROS 2 node, which communicates with the others via the ROS-typical publisher-subscriber model.

### Evaluation procedure

To characterize the behavior of each node when running locally but also remotely, various configurations of the ROS 2 nodes were analyzed. The robot arm controller node and the RealSense camera node could only be run locally, since they interface with the hardware. This left the computer vision and path planning nodes as candidates to be analyzed since they could be offloaded to the server. The evaluation procedure was designed to evaluate every combination of these two nodes on the server and local PC. The resulting four test cases are shown in Fig. 3.

**Fig. 4:**
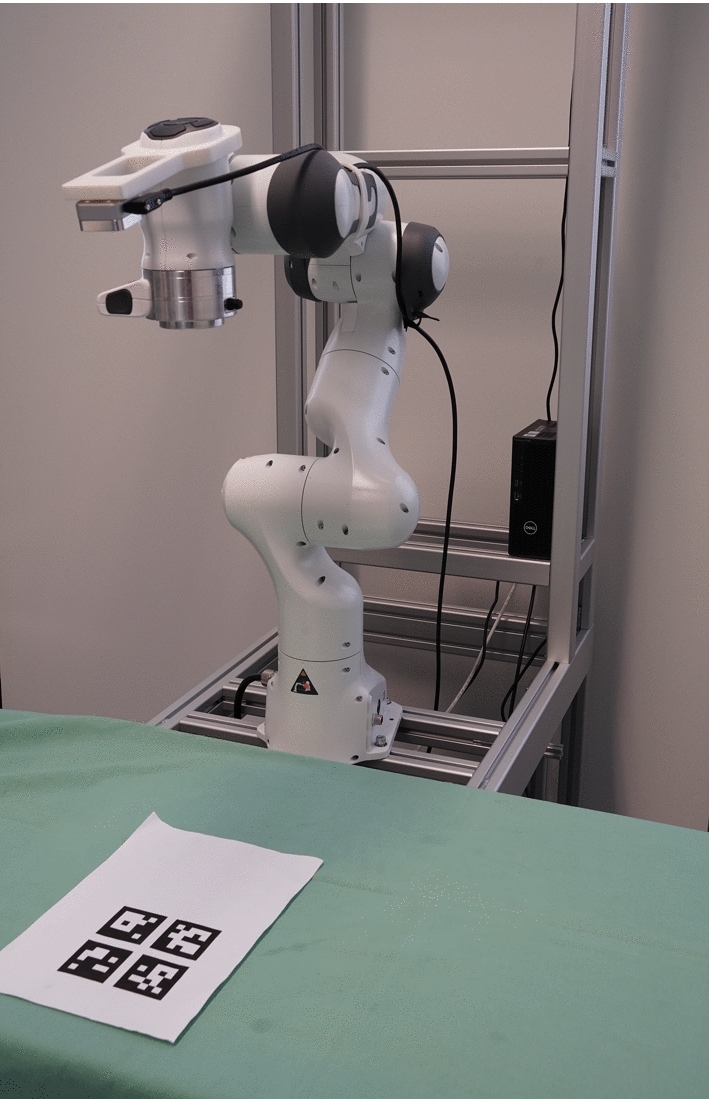
Starting configuration for the evaluation procedure. The robot was always started from this ‘ready’ position to simplify the evaluation workflow. The ArUco target on the table was placed so that it was in view of the hand camera from the ‘ready’ position. It was not moved during the evaluation runs

Before each evaluation run, the robot was positioned in the default ‘ready’ position. The ArUco marker target was placed directly in front of the robot base in view of the end-effector camera. The starting configuration for each trial run can be seen in Fig. 4.

For each configuration, ten evaluation runs were performed. Each evaluation run consisted of commanding the robot to move to the target, resetting to the ‘ready’ position, and then commanding the robot to move to the target again. During each run, all network traffic of both PCs was captured via the tcpdump command line utility [30]. Simultaneously, the CPU usage was reported by the kernel and recorded every second as a percentage of the maximum performance possible by the respective computer via the nmon monitoring utility [31]. In each evaluation run, the CPU monitoring tool was launched before starting the robot task. The remote nodes for the task were then automatically started on the server during the launch process via FogROS2. This step required setup time to create the necessary container and copy the node’s source code before they were able to launch. Once the remote nodes were set up, the VPN connection was automatically established. The network monitoring was started as soon as this VPN setup was complete, just before executing the robot task. Given the large amounts of data sent between the computers, only the first 300 bytes of each packet were captured by tcpdump. This proved to be sufficient since only the Ethernet and IPv4 header of a packet are needed for the latency measurements, the former with a length of 14 bytes in most cases [32] and the latter with a length of 20 bytes [33]. The remaining data provided the ability to discriminate packets with the same headers and eliminate false matches. Upon completion of the evaluation runs, the resulting network and CPU usage data were aggregated and evaluated for each node configuration. The investigated metrics were CPU usage, throughput, and latency. The method of aggregation varied by metric to best highlight the relevant characteristics. The CPU usage was normalized to remove the impact of differing runtime on the CPU averages and plotted as bar charts. The throughput was aggregated by stacking the individual throughput plots. This allowed for an overview of the throughput usage trends over time. Additionally, a box plot of the throughput provided insight into the overall throughput distribution across the individual evaluation runs. The latency data were concatenated and then plotted as a violin plot to highlight the statistical distribution of packet delay for each configuration.

**Fig. 5:**
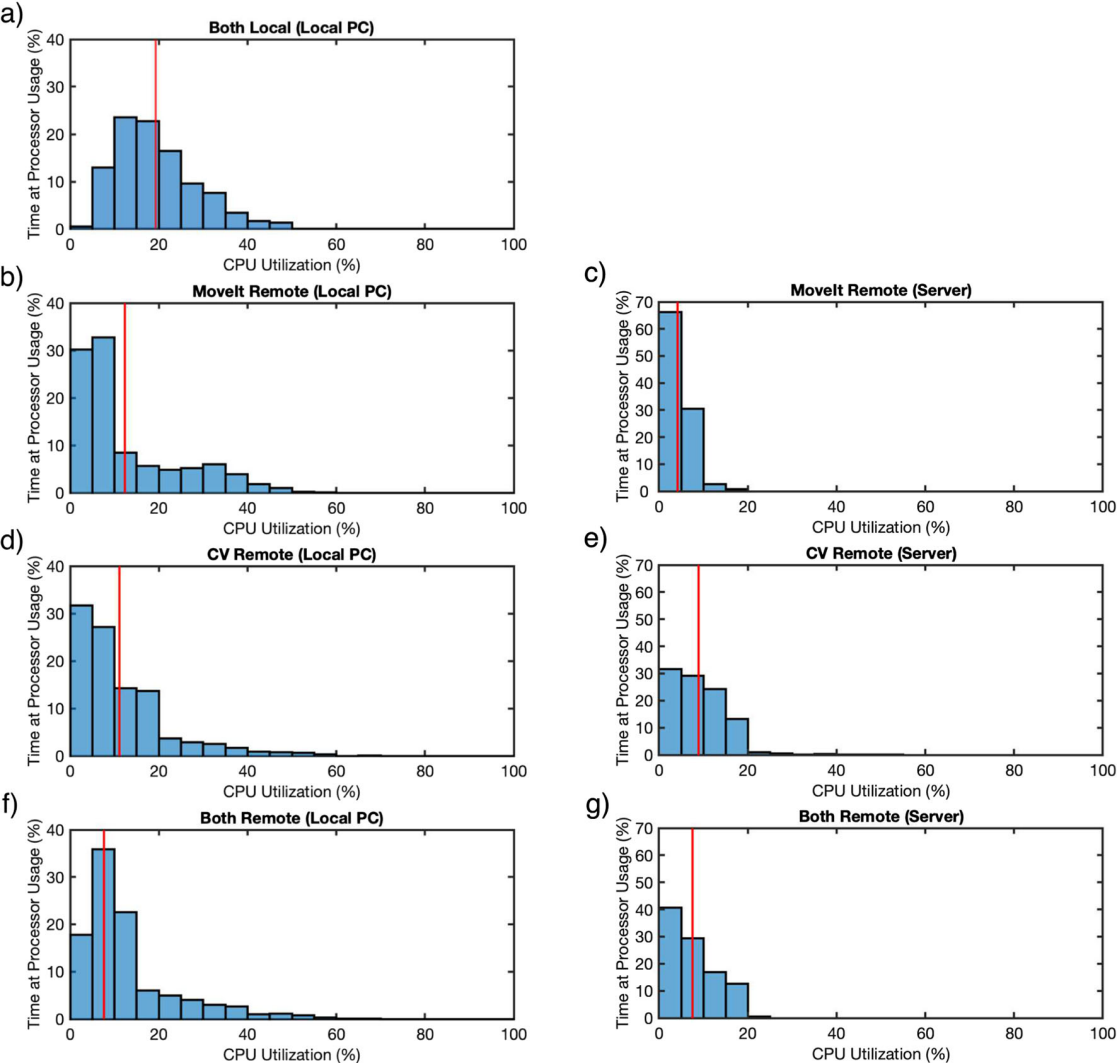
CPU usage of the local PC and server for the different configurations. The red line indicates the normalized mean CPU utilization across all runs for a given configuration; **a** both local configuration; **b**, **c** MoveIt Remote configuration; **d**, **e** CV Remote configuration; **f**, **g** Both Remote configuration

## Results

### CPU usage

Given the server and the local PC’s different CPU thread count (server: 12 threads, local PC: 16 threads) and maximum clock speeds (server: 4.0 GHz, local PC: 2.9 GHz), comparisons between the two computers are not possible based on these data alone. However, one can still make several observations about the effects of each node on the computers individually. The CPU utilization on the local PC follows a predictable trend. As shown in the left column of Fig. 5, running the entire demonstration task locally was the most demanding configuration for the local PC, using, on average, 19.3% CPU. While all configurations that offloaded nodes to the server used less CPU, moving the more computationally demanding CV node to the server reduced the CPU load effectively by the same amount as moving the less demanding MoveIt node. These configurations used, on average, 12.1% and 12.4% of the local CPU, respectively. Offloading both nodes to the server resulted in a reduction of the average CPU load to 12.0%. While the trend generally follows expectations, the actual difference between the remote node configurations is effectively nonexistent. The CPU usage of the server exhibits a similar trend. While moving only the MoveIt node used an average of 4.2% of the server CPU, running the CV node on the server required almost twice as much CPU with an average load of 8.9%. Running both nodes on the server placed an average CPU load of 7.6% on the server, a smaller but similar load to running the CV node alone. The distribution of the server CPU load for each node configuration is summarized in the right column of Fig. 5.

### Throughput

**Fig. 6:**
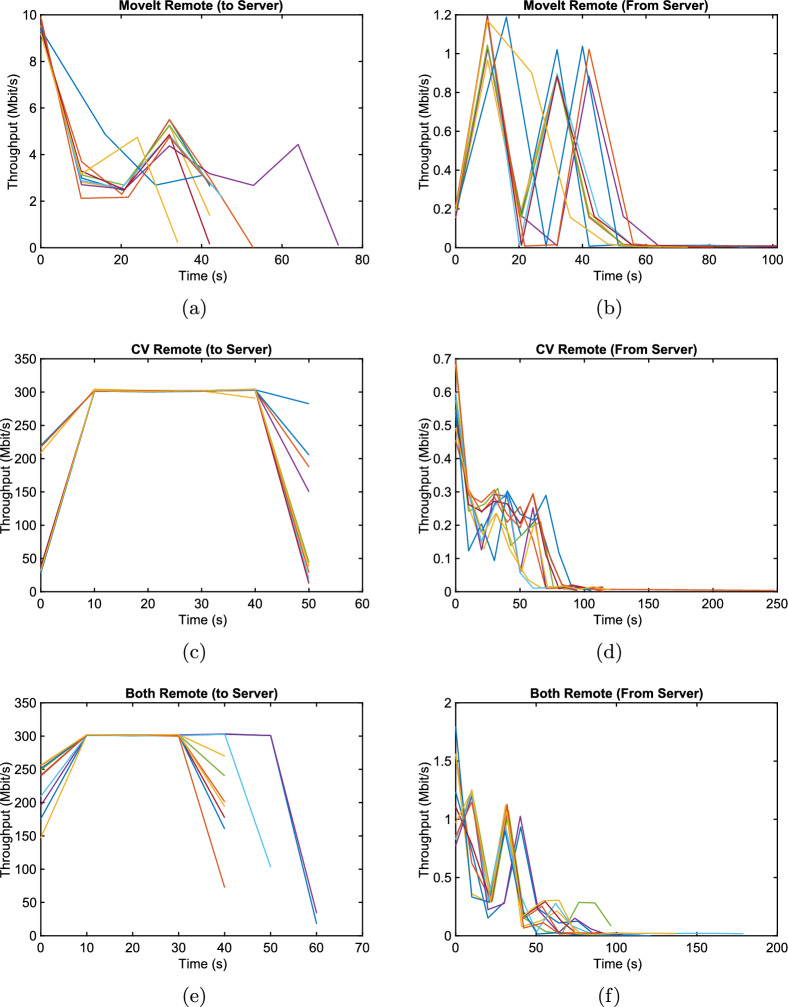
Stacked plots showing the throughput to and from the server for all runs of a given node configuration as a function of time: **a**, **b** MoveIt Remote configuration; **c**, **d** CV Remote configuration; **e**, **f** Both Remote configuration

The throughput of the packets was calculated using a ten-second window, limiting the resolution of the resulting data. Nonetheless, several trends can be identified, as the network traffic resulting from the different node configurations varied considerably. A comparison of the stacked throughput plots for all evaluated configurations is shown in Fig. 6.

With the CV node or both nodes running on the server, the throughput from the local PC to the server was fairly constant at just over 300 Mbit/s during the task. The data sent back to the local PC was comparatively minimal. There are spikes in network activity common across all the CV remote runs. They are quite small, however, ranging from just over 0.2 Mbit/s to just over 0.3 Mbit/s. This was anticipated since the output data of the computer vision node is a pose, which is the smallest message transmitted between nodes. The throughput was significantly lower when only the MoveIt node was offloaded to the server. It was also much more irregular, occasionally spiking when path planning requests and responses were sent, but otherwise dropping even lower. These spikes in traffic to the server peaked at approximately 4.3 to 5.5 Mbit/s. The responses, on the other hand, resulted in spikes between 0.85 to 1.2 Mbit/s. In the case where both nodes were offloaded, the throughput profile to the server was very similar to the CV Remote runs, and the traffic returning to the local PC strongly resembled that of the MoveIt Remote runs. This is as expected since the CV node takes the same image stream it would when running remotely alone, then the two nodes communicate between themselves on the server and only return the final trajectory result, just as the MoveIt node would if only it were remote. As a result, Fig. 6e strongly resembles Fig. 6c, f is very similar to Fig. 6b. They only significantly differ at the beginning of the run, likely due to different starting conditions of the node. As previously mentioned, the random nature of the path planner meant the time taken to execute the robot’s physical movement varied from run to run. Despite the ten-second throughput window used to calculate the throughput, this resulted in some slight shifting of the individual runs in relation to one another. The rough shape, minima and maxima match fairly closely, nonetheless. A comparison of the graphs in Fig. 6b, d, f shows that the cases with the Both Remote and CV Remote runs all start at a peak and steep drop-off in throughput. This could potentially be caused by the traffic required to start the ROS 2 infrastructure since the measurement is started immediately as the robot task starts. In any case, it also likely leads to the elevated whiskers.

**Fig. 7:**
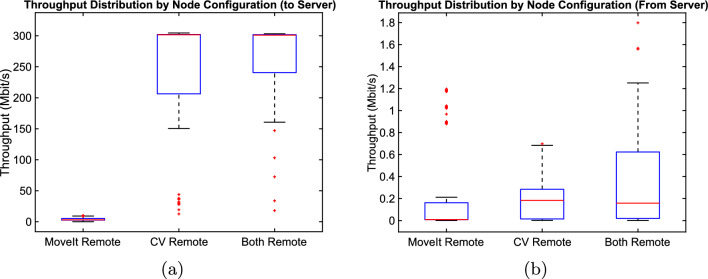
Box plot graphs showing the throughput distribution from: **a** local PC to server, **b** server to local PC. The red line indicates the median, while the red crosses depict outliers in the data set

When looking at the three configurations from a higher-level perspective, one can further identify differences between the nodes’ traffic. The throughput distribution to the server, shown in Fig. 7a, is quite predictable. Streaming images to the remote CV node dominates the network traffic. This is reflected in the mean of the CV Remote and Both Remote cases differing only by 5.8 kbit/s (301.06 Mbit/s for Both Remote and 301.64 Mbit/s for CV Remote.). The MoveIt node’s throughput is significantly less, averaging only 3.11 Mbit/s. This is expected, since only infrequent planning goals are sent. A comparison of the average traffic sent to the local PC for each node configuration is shown in Fig. 7b. The median for the CV Remote and Both Remote runs are very similar at 0.18 Mbit/s and 0.16 Mbit/s. In the MoveIt Remote configuration, the return traffic was much lower, at 0.01 Mbit/s.

### Latency

**Fig. 8:**
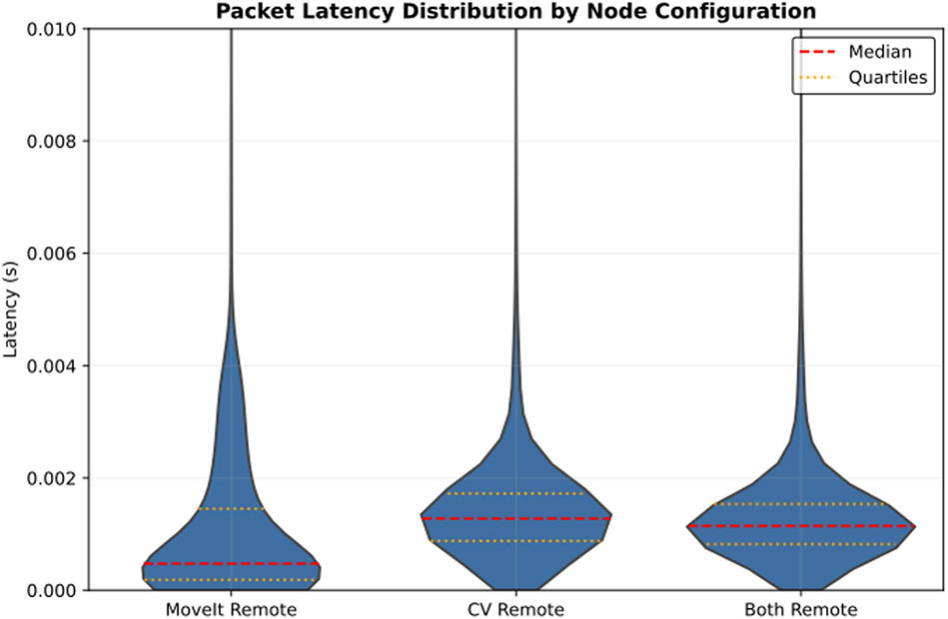
A comparison of the statistical distribution of packet latency for all runs of each configuration. The two types of dotted lines indicate the median and upper and lower quartiles of the distribution. The maxima have been clipped to show more details in the main regions

The latency was calculated for each packet and aggregated across all the runs to enable a comparison of the latency for the different node configurations. Given the essentially stochastic nature of packet latency, plotting the latency as a function of time did not yield relevant insights. Instead, the results of all the runs with a given configuration were plotted as a violin plot to highlight the statistical distribution of the packets in Fig. 8. The traffic between the computers experienced a lower delay when only the MoveIt node was remote. The median delay in this configuration was 0.47 ms. With the CV node remote, the median delay was 1.28 ms, and when both nodes were remote, the median delay was 1.11 ms. The latency maxima followed a similar profile: The MoveIt Remote runs had a maximum delay of 20.14 ms. The computer vision node experienced a maximum latency of 44.50 ms, while both nodes being remote resulted in a maximum of 37.37 ms.

## Discussion

The results of the evaluation clearly show that no superlative configuration exists. Rather, the optimal configuration depends on the hardware and network capabilities, as well as the specific usage scenario. With the use case of a telerobotic examination system in mind, one can nevertheless create a profile of each type of MAF and its characteristics via a qualitative comparison. The local PC’s usage summary (see Fig. 5) clearly shows a reduced CPU load for the local PC when applications are offloaded to a remote server. Curiously, varying which node was run on the server had little effect on the mean local CPU usage. However, the distribution of the CPU usage indicated differences in the usage profiles of the nodes. The cause of this would be interesting to investigate further with a more detailed CPU measurement.

**Table 1:** A table summarizing the behavioral characteristics of the investigated MAFs, used as a basis to generate an allocation strategy

MAF name	Network load	CPU load	Latency requirement	Impact on latency
Camera node	Med/high	Med, constant	Med	Med
MoveIt node	Low	Low, intermittent	Low	Low
CV node	Med/high	Med, constant	Med	Med
Controller node	Low	Med, constant	High	Low

**Table 2:** The proposed allocation strategies of the investigated MAFs based on their measured behaviors

MAF name	Allocation strategy
Camera node	Required to run on local PC due to interfacing with hardware, statically allocated
MoveIt node	Unconstrained, run anywhere in network, instantiate on-demand
CV node	Run statically allocated close to edge
Controller node	Currently must run on local PC, could conceivably be offloaded to network with future development, statically allocated

The investigated network aspects also gave other insights into the behavior of the different MAFs. The MoveIt node only requires intermittent throughput capacity when receiving a request or sending a result. Even then, the total throughput used is quite minimal. If the path planning were to become more complex and, as a result, more of a computational burden, it would be an ideal candidate to be offloaded from the local PC, since even a fairly complicated motion planning problem can be represented as a compact request, and the resulting trajectory also does not necessarily grow with increasingly complex boundary conditions. Placing such an MAF with varying computational demands and low throughput on resources within the network would therefore achieve a reduction in CPU load for the local PC at a cost of only a small increase in bandwidth. The CV node, in comparison, requires a fairly constant, relatively large amount of bandwidth, analogous to its CPU use. While offloading such a type of MAF to a server provides a consistent baseline reduction for the duration of the robot task, it comes at the cost of significant network utilization. These bandwidth limitations restrict the use of this configuration only to scenarios where a very good network connection can be guaranteed, for example, within a clinic or between larger cities. While it is possible to use compression to reduce the required bandwidth, this again comes at the cost of computation and latency.

In cases where the network connection is not as capable or in cases where many other devices wish to communicate via the same network, this high, constant data use could become a significant concern. This challenge does, however, highlight one of the main advantages of the fog computing architecture: instead of transmitting the data all the way to a centralized cloud, it can be offloaded to resources at the network edge, thereby reducing the distance the data must travel, improving latency, and limiting the impact on the rest of the network. In the evaluated system, these limitations do not impact the performance since it currently runs in nearly ideal network conditions. However, they could become more relevant in more complex network scenarios. The system performance under realistic conditions, i.e., in congested or degraded networks, should be investigated in future work.

Similar conclusions can be drawn for latency. While the network latency experienced by all demonstration task nodes is very close to the 1 ms latency target of 5G Ultra-Reliable Low Latency Communication (URLLC), providing a good starting point for further development of this technology for the 6G standard, some preliminary test runs during the development phase showed that the URLLC goal latencies cannot always be guaranteed in cases of higher network utilization. A summary comparison of the investigated nodes based on all these factors is shown in Table 1.

With the characterization of the various requirements and loads placed on the underlying system, one can develop preliminary strategies to position such MAFs within a distributed robotic system. A constant, high-throughput MAF like the CV node, for example, if offloaded, should be delegated to a DU relatively close to the robot to minimize latency and reduce the number of network links affected by the high data throughput. Furthermore, given its constant base load, it is effectively impossible to do anything other than run it in a static container for the duration of the robot task run. This could still provide a benefit, however. In a hospital scenario, one could place a server on the edge of the clinic network, running the CV nodes for multiple telerobotic examination systems, provided the network could handle the necessary bandwidth. A qualitative observation that may affect the allocation strategy of the CV node is that the trajectories generated with only the CV node remote were much worse than in other configurations. The CV remote evaluation had to be aborted much more frequently due to imminent collision or joint limit violations than the evaluation runs for other node configurations. The cause for this may be related to the seeding of the random path planning algorithm, and replacing RRT-Connect with a more systematic planner should eliminate this problem. An intermittent, low-throughput MAF like the MoveIt task should be offloaded differently, given its more relaxed constraints. For one, latency is not the limiting factor in the time between sending a path planning request and receiving a response, and the bandwidth required to do so is minimal. Therefore, such an MAF could more easily be delegated anywhere in the network. Furthermore, as it only runs on-demand, the MoveIt node could launch to generate the requested trajectory and then immediately be terminated. This could also allow such an MAF to be delegated to wherever the computational load is lowest in the network each time it is needed. On top of this, rather than constantly running an idle MAF for each telerobotic examination system, the number of nodes could be dynamically scaled up and down as the current demand requires, and allocated in the network to anywhere computing capacity is available. The more relaxed constraints on this MAF’s deployment make it an ideal candidate for exploring more dynamic forms of fog computing. In a hospital scenario, the MoveIt nodes could be run in many parts of the network, including off-premises. One critical consideration for this on-demand allocation is that one would need to be able to quickly launch this node; otherwise, the starting time of the remote node would become the bottleneck of the path planning pipeline. These allocation strategies are summarized in Table 2.

The aim of this work is to provide a foundation for the development of network-based medical robotics applications. Consequently, the demonstration task used for evaluation was designed to be as simple as possible while still preserving the characteristics of different types of MAFs. While the task pipeline alone may not yet enable a full telemedical examination, the evaluation results are generalizable to other MAFs with similar requirements on the network and computing infrastructure. In future work, the proposed allocation strategies should be taken into account when expanding to more sophisticated control pipelines or complex network setups.

## Conclusion

6G communication networks are envisioned to fulfill the stringent requirements of emerging healthcare applications such as robotic telemedicine. In this paper, we use a telerobotic examination system consisting of various MAFs running on distributed hardware, enabling the flexible implementation of various software configurations. To investigate the potential of distributed computing scenarios, such as fog computing, in telemedical applications, we offloaded different combinations of nodes from a simplified demonstration task to a network server and analyzed the performance and resulting network traffic of the telemedical application. Based on these results, we developed allocation strategies for the nodes, representing different types of MAFs. This work lays the foundation for the development of medical robotic applications using 6G network architectures and emerging types of distributed computing scenarios in particular. In the future, we plan to investigate the system using a more comprehensive task pipeline, as well as the capability to dynamically shift MAFs within the network based on current situational demand, which could help to further optimize the performance of network-based medical applications.
